# Genome engineering on size reduction and complexity simplification: A review

**DOI:** 10.1016/j.jare.2023.07.006

**Published:** 2023-07-12

**Authors:** Xiang-Rong Chen, You-Zhi Cui, Bing-Zhi Li, Ying-Jin Yuan

**Affiliations:** Frontiers Science Center for Synthetic Biology and Key Laboratory of Systems Bioengineering (Ministry of Education), School of Chemical Engineering and Technology, Tianjin University, Tianjin, China; Frontiers Research Institute for Synthetic Biology, Tianjin University, Tianjin, China

**Keywords:** Genome simplification, Size reduction, Complexity simplification, Synthetic biology

## Abstract

**Background:**

Genome simplification is an important topic in the field of life sciences that has attracted attention from its conception to the present day. It can help uncover the essential components of the genome and, in turn, shed light on the underlying operating principles of complex biological systems. This has made it a central focus of both basic and applied research in the life sciences. With the recent advancements in related technologies and our increasing knowledge of the genome, now is an opportune time to delve into this topic.

**Aim of review:**

Our review investigates the progress of genome simplification from two perspectives: genome size reduction and complexity simplification. In addition, we provide insights into the future development trends of genome simplification.

**Key scientific concepts of review:**

Reducing genome size requires eliminating non-essential elements as much as possible. This process has been facilitated by advances in genome manipulation and synthesis techniques. However, we still need a better and clearer understanding of living systems to reduce genome complexity. As there is a lack of quantitative and clearly defined standards for this task, we have opted to approach the topic from various perspectives and present our findings accordingly.

## Highlights

• Genome simplification can promote the understanding of the law of life, which can be divided into two aspects: reducing genome size and simplifying complexity.
• Shifting focus from a trial-and-error approach towards a more systematic analysis and thoughtful design for genome size reduction is necessary.
• SCRaMbLE, a synthetic chromosome rearrangement technology, enables large-scale simplification of synthetic chromosomes and is a key avenue for future simplification research.
• Genome complexity reduction represents a form of genome simplification that necessitates a comprehensive understanding of living systems.
• Establishing a metric for genome complexity is essential and urgent.

## Graphical abstract

**Figure:**
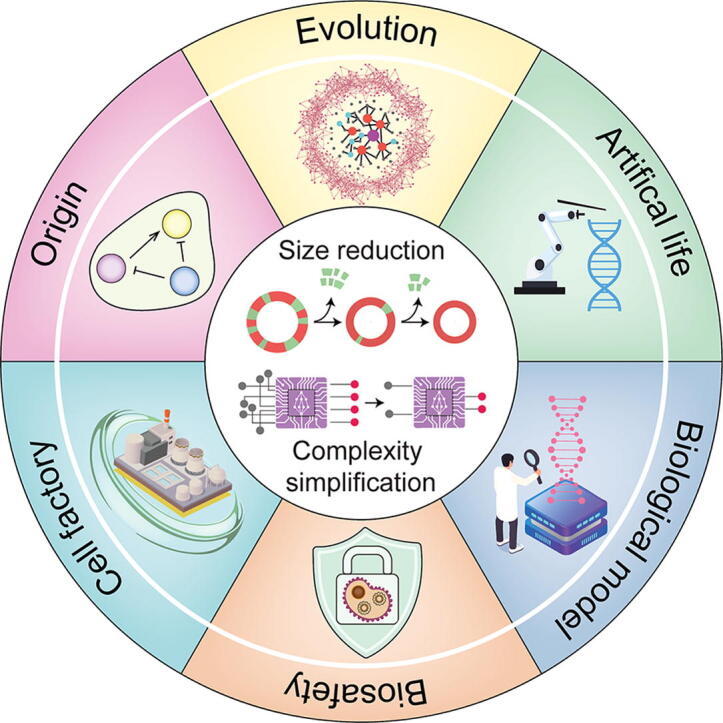

## Introduction

**Fig. 1:**
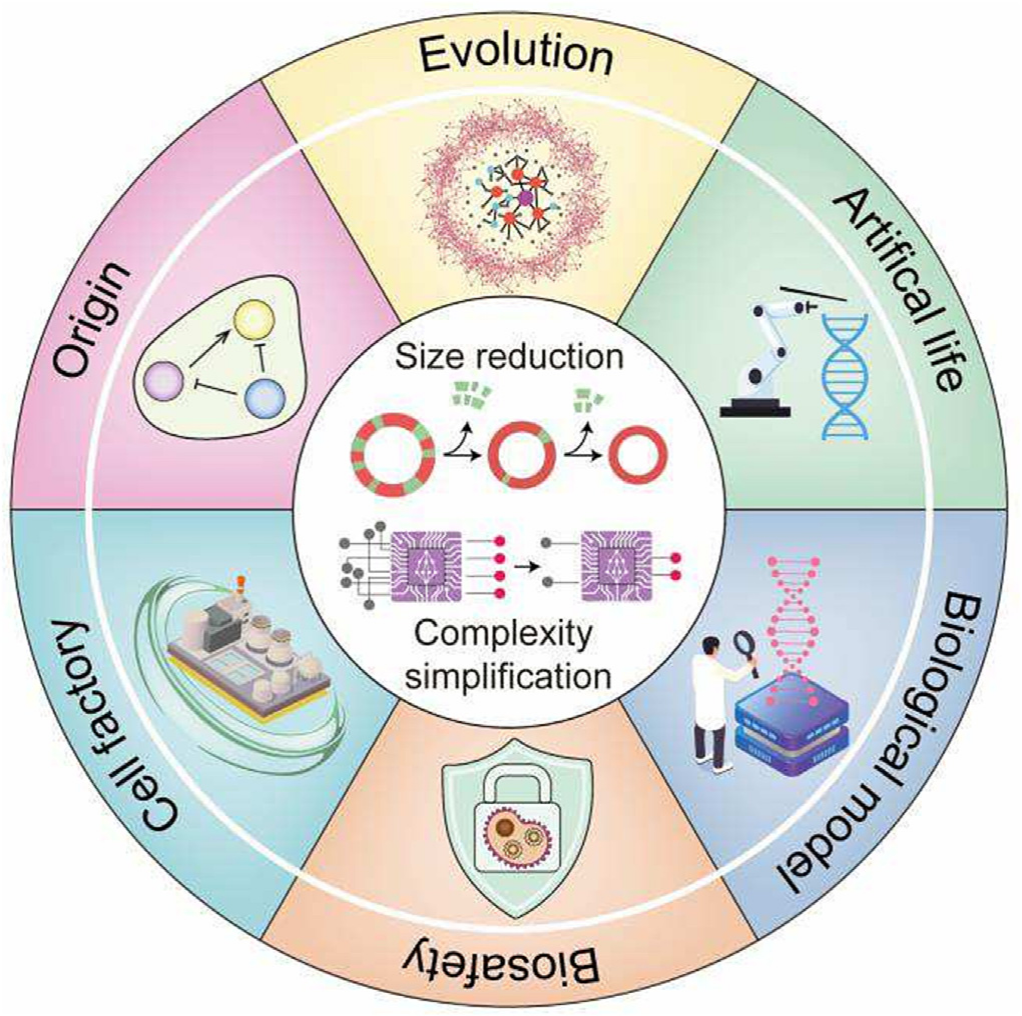
Schematic diagram of genome simplification. The simplification of the genome involves two perspectives: size reduction and complexity simplification. This simplified genome can be employed to investigate scientific inquiries, such as the origin of life, evolution, and artificial life. And it can have practical applications, such as building biosafety genomes, cell factories, and biological research models.

Genome simplification is a significant topic in the field of life sciences that has garnered attention from its conception to the present day. Exploring the problem of genome simplification can provide insight into the essential compositions of the genome. And it is a central focus of both basic and applied research in the field of life sciences (Fig. 1). On the one hand, the complexity of the genome is reflected in the fact that it is composed of an increasing number of genomic elements with progressively intricate interactions. On the other hand, these genomic elements provide the basis for the development of more complex laws of life. Therefore, genome simplification encompasses two aspects (Fig. 1). Firstly, it involves minimizing unnecessary DNA sequences and identifying the minimum set required for genome constitution [1], [2], [3]. Secondly, it aims to create simpler life forms [4]. Genome simplification serves as an excellent platform for basic research on scientific questions such as the origins of life [5], genome evolution [6], and gene interactions [7]. It promotes understanding in the field of life sciences [8] and inspires artificial life [9], [10]. In terms of applied research, the simplified genome can be used to construct industrial chassis cells [11], [12], [13] and biological research models [7], [14], [15], [16].

Numerous research groups are currently exploring the minimal set of active genomes. In Japan, the “Minimum Genome Factory” (MGF) project was launched in 2001. Several model microorganisms have had their genomes reduced in this project. Researchers at the J. Craig Venter Institute (JCVI) have been leading the way in synthetic genomics and minimal genome research [14], [17]. In 2016, Venter's team constructed a functionally minimal synthetic *Mycoplasma* genome, which is the smallest autonomously surviving genome reported to date [14]. The Sc2.0 project aims to synthesise all 16 chromosomes of *Saccharomyces cerevisiae*, making it the first eukaryotic genome synthesis project and a major platform for simplifying eukaryotic genomes. In 2020, the Sc3.0 plan was proposed based on the Sc2.0 plan to cluster essential genes for further genome reduction [18].

While reducing the size of genome can sometimes be a means of reducing its complexity, the type of complexity simplification we are discussing here is focused on streamlining other principles of life. In this field, many research teams have made significant pioneering advances. In terms of codon usage, researchers are exploring simpler codon usage schemes [19], [20], [21]. In 2016, experts in the field of synthetic biology initiated the GP-write project, which aims to challenge the rewriting of the human genome [22], [23]. Researchers are also exploring the simplest form of chromosome number, which provides a new way to study the relationship between chromosome structure and function [24], [25]. Additionally, in the realm of genomic repetitive elements, researchers are simplifying the functional repetitive DNA elements such as tRNA genes and rRNA and investigating the significance of their multi-copy existence.

We will discuss genome simplification from two perspectives: the first starts with minimal genome composition, which refers to fewer genes and expressed proteins; the second perspective focuses on simplifying complexity, which means making the genome simpler, more predictable, and more controllable. In our article, we summarize the advancements in genome simplification achieved in two aspects and explore potential future directions.

## Reducing genome size

The basic principle of genome reduction is to identify and eliminate non-essential elements. In this section, we use the term “non-essential elements” to describe both non-essential genes and non-coding sequences that are not necessary.

### Identification of non-essential elements through experimental analysis

Obtaining essential data through scientific experiments and analyzing its underlying principles are effective methods for gaining knowledge. Global Transposon Mutagenesis is widely utilized due to its ease of operation and host flexibility [17], [26], [27], [28], which spurred the initial efforts to identify non-essential genes in bacteria and eukaryotic microorganisms, and remains the primary method for determining non-essential genes. In 1999, this method was used to identify between 265 and 350 protein-coding genes required under laboratory conditions [26]. Since then, this technique has been widely employed in various bacteria [17], [27], [28] and fungi [29]. However, since transposon insertion is a random process, certain sequences may tolerate it, which could lead to a misjudgment of essentiality. Constructing a knockout library targeting specific sequences can provide a complementary approach [30], [31], [32]. Researchers systematically create single-gene knockout libraries of various cells, providing valuable resources for functional genomics research.

**Fig. 2:**
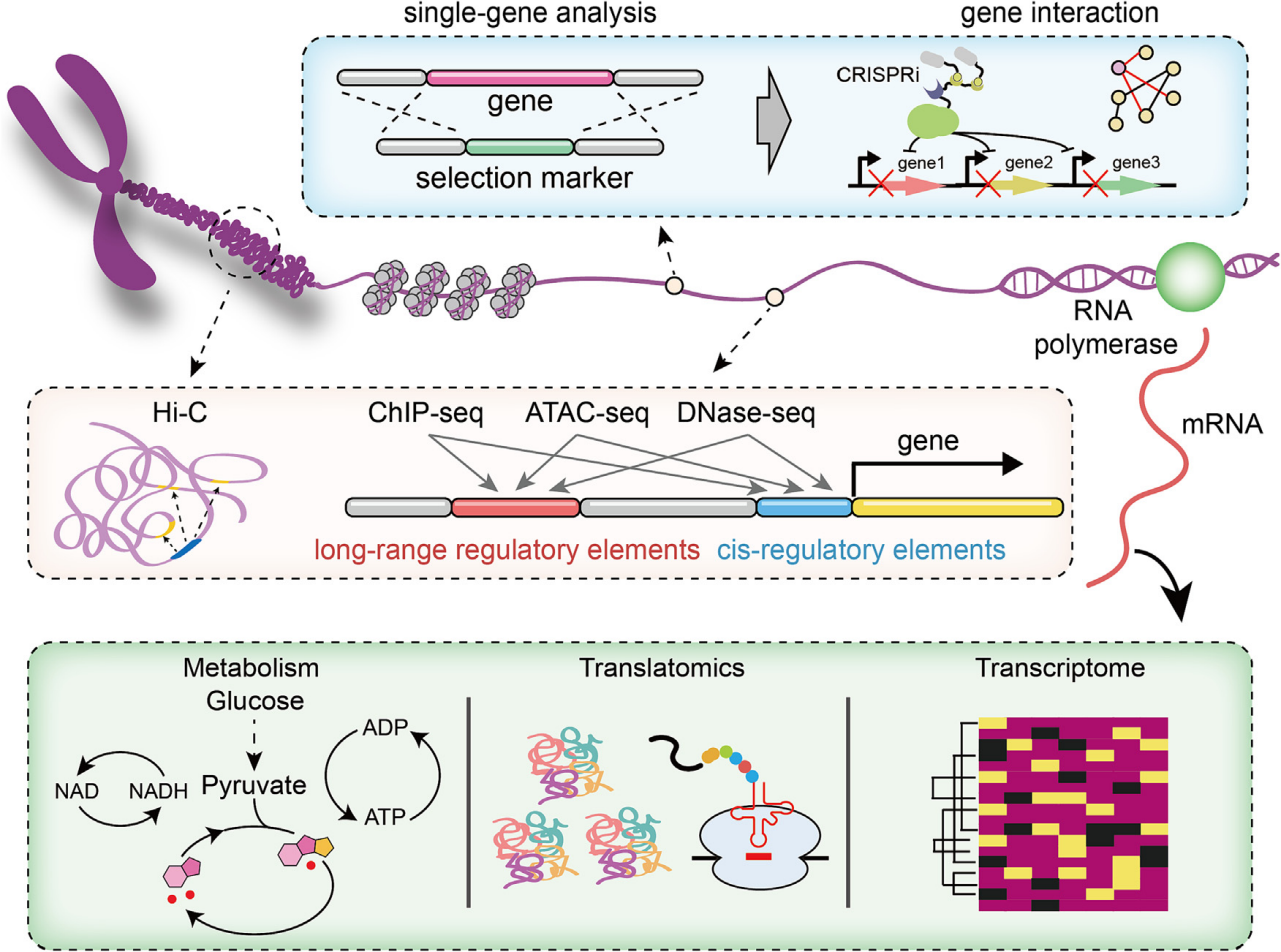
A systemic perspective to identify and understand non-essential elements.

The proportion of non-coding sequences in bacterial genomes is relatively insignificant, so the main challenge in reducing bacterial genomes is to identify non-essential genes. As redundant sequences are gradually reduced, gene interaction relationships assume greater significance. Hence, it is imperative to thoroughly explore gene interaction relationships and devise a systematic scheme for genome reduction. The advancement of transposon technology has facilitated the analysis of genetic interactions among multiple genes [33], and the identification of essential genome components with greater accuracy [34], [35]. Nevertheless, some constraints remain in the analysis of particular gene combinations and the creation of transposon systems in higher organisms. The advancement of genome editing technology, particularly CRISPR, has significantly enhanced the ability to mine DNA functions on a genome-wide scale in both non-model organisms and higher eukaryotes. Researchers have successfully constructed the CRISPRi system using dCas9, which has been extensively demonstrated to possess multi-site transcriptional regulation capabilities [36], [37]. At present, genome-scale CRISPRi libraries have been established for both human and mouse genomes, and researchers have screened K562 cells for gene essentiality [38]. By utilizing CRISPRi to inhibit the expression of each essential gene in *Bacillus subtilis*, the interaction relationships between essential genes in vivo can be uncovered [39]. These efforts provide a framework for the systematic study of gene function (Fig. 2).

Higher eukaryotes contain a significant number of non-coding sequences [40], some of which are known as genome regulatory sequences. Efforts to reduce complex genomes have focused on identifying the functional non-coding sequences from the vast number present in such genomes. To understand the function of non-coding sequences and establish a catalog of functional non-coding genomic elements, researchers employ various measurement methods (Fig. 2) such as ChIP-seq, ATAC-seq, DNase-seq, and Hi-C.

### The prediction of non-essential genes

The status of non-essential genes is not static; instead, it is influenced by both environmental conditions and genetic background [41]. Therefore, the combination of so many variables cannot be exclusively confirmed through experiments, and computer-aided identification of non-essential genes holds practical significance.

Bioinformatics has played a crucial role in the prediction of essential and non-essential genes. By using known information and biological characteristics of essential genes, combined with computer science and various mathematical algorithms, accurate prediction models can be established to rapidly identify the necessity of genes. A prediction model was constructed, utilizing gene ontology enrichments and KEGG pathways, resulting in nearly flawless performance (Matthews correlation coefficient = 0.951) in discriminating between essential and non-essential genes [42]. Arun *et al.* [43] conducted a study of the relationships between gene conservation, duplication, constitutive expression, and gene essentiality in *Escherichia coli*, demonstrating their intricate interrelationship and strong predictive power for essentiality. Global analysis of protein interaction networks can effectively reveal the relationships between genes, showing that essential genes have a higher degree of connectivity and generally have more interactions than non-essential genes [44]. Using only sequence information, researchers propose a deep neural network architecture that accurately and efficiently predicts the essentiality of microbial genes [45]. Machine learning predicts gene essentiality by leveraging known information, such as genome-scale metabolic networks [46], intrinsic attributes (statistical and physicochemical data) [47], or comprehensive publicly available genomic-phenomic datasets [48].

The functional redundancy of multi-copy genes enables their reduction to a certain extent. Identifying and analyzing these genes provides valuable insights into genome reduction. Through bioinformatics analysis, researchers thoroughly examined the evolutionary relationship of *Sox* family genes and successfully identified multiple copies within this gene family [49]. Notably, rRNA and tRNA serve as prominent examples of multi-copy genes. Several techniques, based on sequence alignment, have been developed to aid in the search and identification of these gene types [50], [51], [52].

Identifying gene copy numbers in complex genomes requires advanced genome sequencing techniques and data analysis capabilities. To meet these challenges, researchers have developed several bioinformatics analysis methods, including Parascopy [53], CloneCNA [54], and SG-ADVISER CNV [55], for accurate detection and analysis of copy number variations.

The discovery of natural tiny genomes can greatly aid in determining the core genetic functions of organisms. Researchers have observed that certain microorganisms, which rely on a host for survival, have streamlined their genomes to less than 1 Mb [56], resulting in the loss of numerous genes related to biological synthesis and substrate degradation. Since 2006, the genomes of obligate symbionts of insects have been discovered in independent lineages, with sizes less than 300 Kb [56]. The smallest genome discovered thus far is that of *Hodgkinia cicadicola*, a symbiont of cicadas, with a size of only 144 Kb [57]. Typically, these tiny genomes encode only the most fundamental functions of the organism.

Comparative genomics reveals core genes that are conserved across evolutionarily distant species and sheds light on the emergence of redundant components [58], [59]. It can significantly narrow down the scope of non-essential genes. For instance, the identification and removal of genomic K-islands exemplifies the use of comparative genomics in minimizing genomes.

We need to not only identify redundant rules within natural genomes, but also extract useful information from a vast array of artificially reduced genomes. A paradigm for investigating the minimal genomes based on multi-omics data is currently emerging [4], [60], enhancing our comprehension of redundant components (Fig. 2). The comprehensive characterization and joint analysis of *M. pneumoniae*, a genome-reduced bacterium, across its proteome [61], transcriptome [62], and metabolome [63], presented in a series of three papers, represents a significant milestone in the study of a single species using a holistic systems perspective. The genome reduction algorithm [64], [65] and related models [15], [65], [66], [67], [68] provide predictive solutions for streamlining genome efforts. Additionally, machine learning has the potential to predict essential genes in eukaryotic genomes [69], [70] and provide evolutionary predictions for bacterial metabolic systems [71], both of which show great potential in genome streamlining.

As researchers gain a deeper understanding of living systems, the models they build become more realistic. By continually refining algorithms and models, it may be possible to use computers to design the minimal living organism.

### Redefining the non-essential elements

As we delved deeper into the genome reduction, we came to realize that the definition of non-essential elements is not as straightforward as we initially thought. For instance, we must consider the effects of alterations in the genetic background as well as changes in the external environment on gene requirements [41].

A prime illustration of this concept is the phenomenon of synthetic lethality [72], [73]. The occurrence of synthetic lethality elevates the significance of non-essential genes and makes genome reduction more challenging. Aside from genetic background, the environment also plays a crucial role in determining the essentiality of genes. It can be argued that considering the environmental context is a prerequisite for discussing the essentiality of genes [74]. Recent research has revised the traditional binary classification of gene essentiality into four gradients: no essentiality, low essentiality, high essentiality, and complete essentiality [41]. This new classification scheme accounts for the impact of the environment on the essentiality of genes, challenging the conventional notion of non-essential as static. Furthermore, the researchers created single-knockout libraries of diverse genomes and evaluated the performance of the individual knockout strains under various environmental conditions, providing concrete evidence for the environmental dependence of non-essential genes [75], [76].

From an evolutionary perspective, the number of non-essential genes in the genome is dynamically changing [77]. The researchers coined the term “persistent genes” to refer to genes that are present in over 85% of the genomes within a clade [78], [79]. When genes are classified based on persistence and essentiality, it becomes apparent that although most persistent genes are not currently deemed essential, their continued existence implies that their loss would result in lethality or severe negative consequences. Therefore, from a long-term evolutionary perspective, these genes are indispensable.

The human genome is incredibly vast, containing almost three billion bases, and the majority of the sequences remain unknown to us. The systematic mapping of various genome regions by the Encyclopedia of DNA Elements (ENCODE) project has allowed for the assignment of biological functions to 80% of the human genome, which provides us with a relatively rich annotation of non-coding sequences [80]. This update greatly improves our understanding of non-essential genomic elements.

### Reducing non-essential elements

**Fig. 3:**
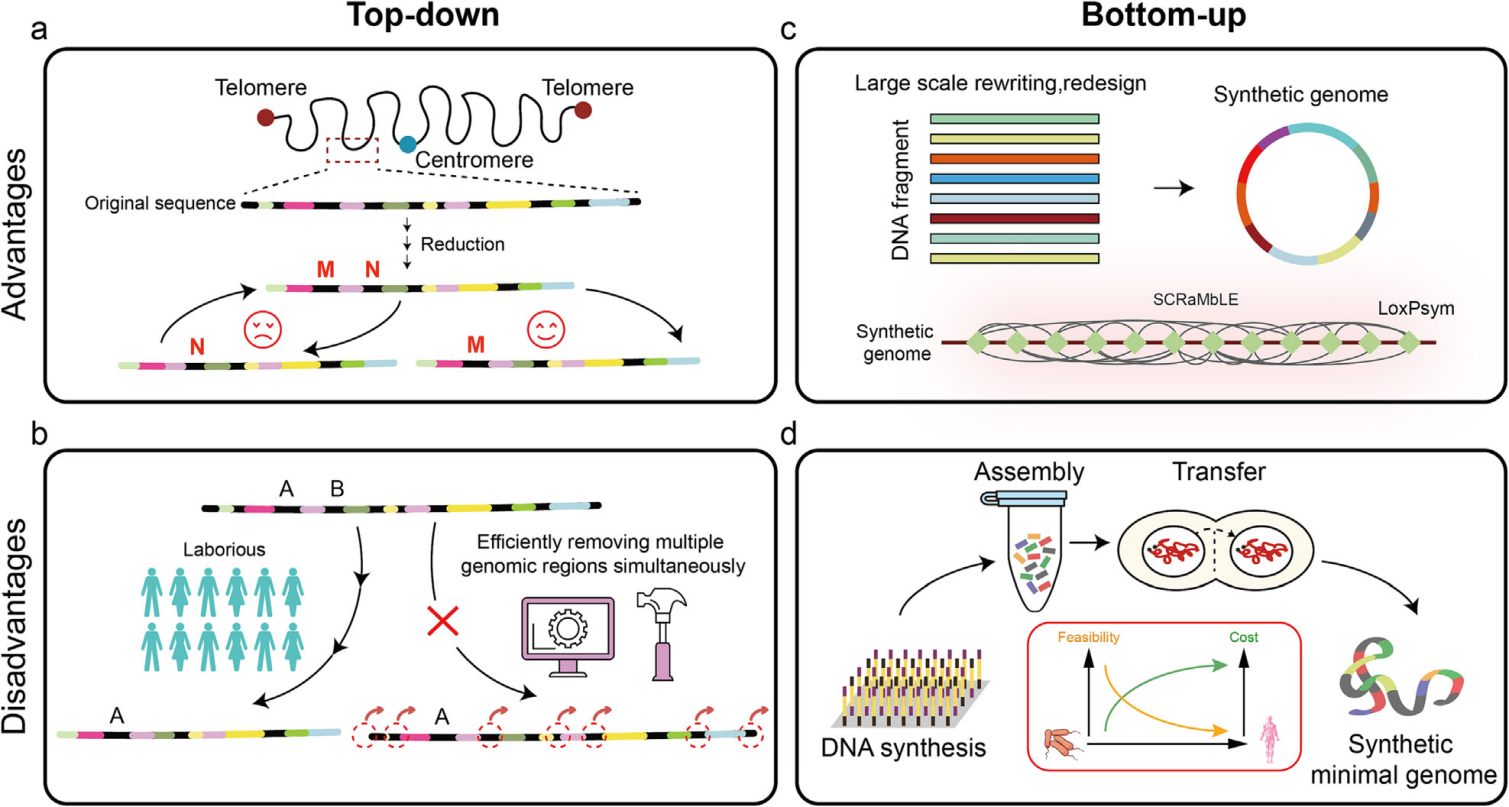
Schematic diagram of genome reduction. There are two strategies for size reduction: top-down and bottom-up, each with advantages and disadvantages. (a) The top-down genome reduction strategy has the advantage of being low-cost and allowing for trial and error. (b) Its current linear process consumes a significant amount of time and energy, which is a disadvantage. (c) The bottom-up strategy offers the benefit of introducing large-scale genomic changes and generating a considerable number of genome collections quickly, simultaneously. (d) It has certain drawbacks in complex genomes, including explicit design principles, expensive synthesis costs, and technical bottlenecks in assembly and transfer.

The reduction process for non-essential elements has yielded two strategies: top-down and bottom-up. When there is insufficient understanding of the genome, the top-down strategy for genome minimization is widely used due to its low cost and trial-and-error nature (Fig. 3a).

Top-down genome reduction involves sequentially removing unnecessary genome sequences. The main advantage of this approach is that it starts with an active genome, meaning that any negative effects caused by sequence deletion can be addressed by reverting to a previous version of the genome. Numerous strains with reduced genomes have been engineered in *E. coli*, ranging from 200 Kb to 1.6 Mb, representing 4.3% to 39% of the original genome [27], [81]. Additionally, up to 36% of the sequences were condensed in *B. subtilis* [82]. The eukaryotic genome contains numerous regulatory sequences and intricate structures, which makes the process of reduction challenging and accounts for the relative scarcity of related works. Researchers were able to reduce the size of the genome of *S. cerevisiae* by 5% using chromosome splitting and loss techniques [83]. In *Schizosaccharomyces pomb*, the LATOUR technique removed a large terminal region (totaling 657.3 Kb) on chromosomes I and II, resulting in a 5.2% reduction of the genome [84].

However, the top-down approach to reduction has certain limitations. No current genome manipulation tool can efficiently remove distinct genomic regions simultaneously (Fig. 3b). Synthetic genomes can be created from scratch [85], unlike genome engineering techniques that modify existing genomes in living cells. This eliminates the limitations imposed by the ability to edit pre-existing DNA and allows for larger scale changes in the genome [86]. Therefore, the bottom-up approach represented by genome synthesis presents itself as a viable alternative. This approach not only enables the removal of numerous non-essential DNA sequences during genome synthesis, but also could incorporate tools for further genome streamlining into the synthetic genome, creating an excellent platform for reduction (Fig. 3c). In 2016, researchers reduced the full chemical synthetic genome of *M. mycoides* from 1,079 Kb to 531 Kb in JCVI-syn3.0, achieving a reduction of 51% of its original genome [14]. This milestone work marks a significant advancement in genome reduction for bacteria. That same year, the researchers also assembled MGE-syn1.0, a 1.03 Mb *E. coli* genome inside budding yeast. This included 449 essential genes and 267 important growth genes, resulting in a genome reduction of 74.1% [87]. However, it has not yet been verified whether the genome can survive on its own. In 2014, Sc2.0 achieved a significant milestone by successfully synthesizing the first yeast chromosome, synIII [88]. The designed synthetic yeast genome became nearly 8% smaller than the native yeast genome by removing sequences like retrotransposons, introns, and LTR repeats [89]. The synthetic yeast chromosome contains loxPsym sites on either side of non-essential genes, making it possible to remove the non-essential genome later using the developed SCRaMbLE tool [88], [90], [91], [92], [93], [94]. This platform enables the study of intricate interactions between non-essential genes, offering unlimited possibilities for genome reduction [95], [96], [97], [98]. Currently, essential genes are dispersed throughout the chromosome, which results in high cell lethality when they are deleted during SCRaMbLE, limiting the utility of this technique for genome streamlining. Nevertheless, the potential of SCRaMbLE for genome minimization will be optimized as essential gene clustering advances [18], [97] and more synthetic chromosomes are achieved.

Of course, there are limitations to the bottom-up approach for genome reduction (Fig. 3d). Firstly, the genomes of humans and higher eukaryotes are too extensive to be synthesized with our current technology. Secondly, our knowledge of non-model organisms and higher eukaryotes is insufficient to enable us to directly design the active genome. Therefore, the development of a flexible and manipulable synthetic genome would be a practical solution for achieving genome minimization through a bottom-up approach. We present Table 1, which summarises the main results of both top-down and bottom-up genome reduction.

**Table 1:** List of major achievements in genome size reduction.

Strain	Strategy	Designation^a^	Original Genome	Deletion	References
*E. coli* (MG1655)	Top-down	△33a	4.64 Mb	1,806 Kb (38.9%)	[81]
*B. subtilis* (168)	Top-down	MGP254	4.22 Mb	1,490 Kb (35.3%)	[167]
*S. cerevisiae*	Top-down	–	12 Mb	600 Kb (5%)	[83]
*S. pombe*	Top-down	A8	12.57 Mb	657.3 Kb (5.2%)	[84]
*S. cerevisiae*	Bottom-up	-^b^	12 Mb	960 Kb (8%)	[89]
*M. mycoides*	Bottom-up	JCVI-syn3.0	1.08 Mb	548 Kb (51%)	[14]
*E. coli* (MDS42)	Bottom-up	MGE-syn1.0^c^	3.98 Mb	2,950 Kb (74%)	[87]

a The names of simplified strains in the original article. ^b^ The deletion is planned but has not yet been completed. ^c^ Not capable of self-survival.

## Reducing complexity for improved understanding

**Fig. 4:**
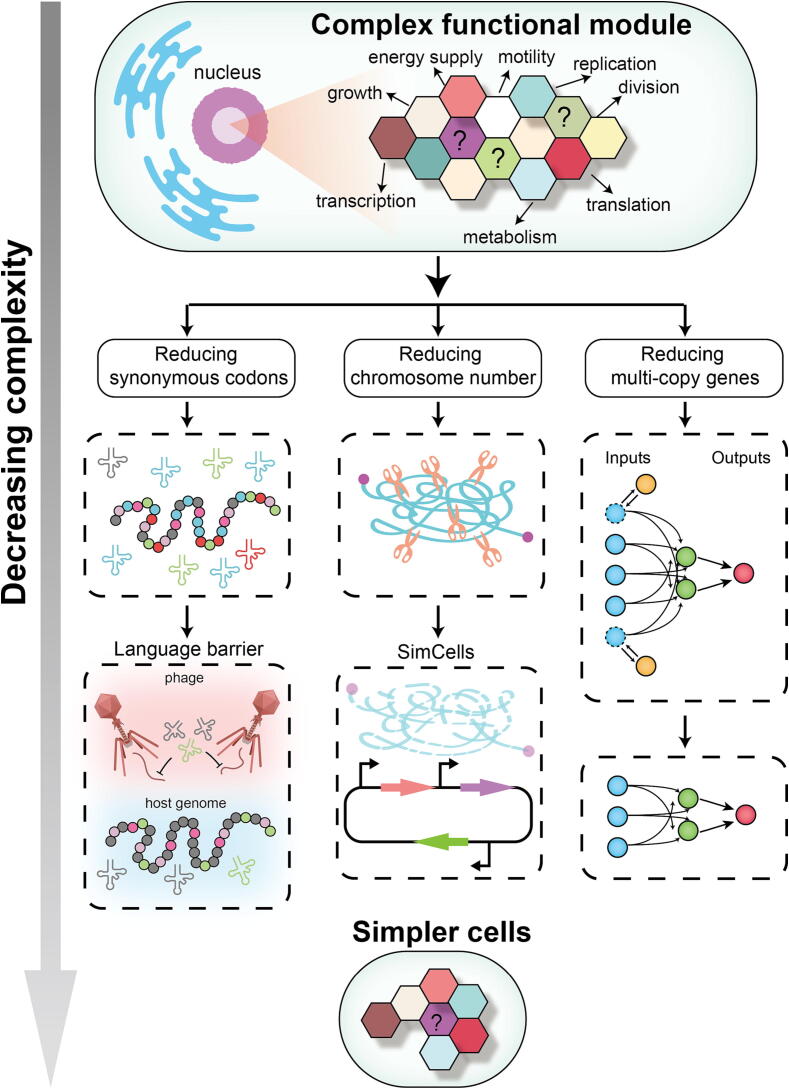
Schematic diagram of complexity simplification. Simplifying synonymous codon usage, reducing the chromosome numbers, and removing genome multi-copy elements might be some strategies for building simpler life forms.

The construction of simpler life forms is one of the objectives of complexity simplification endeavors. In this section, our focus is on simplifications related to codon usage, chromosome numbers, and genome multi-copy elements (Fig. 4).

### Codon recoding: reducing synonymous codons

Synonymous codons are intricate, and synonymous substitution of these codons presents a challenge to the well-written genetic code. Nevertheless, this challenge holds significant theoretical and practical value, including improving our understanding of the genome [19], [20], [21], identifying new regulatory elements [99], and developing strategies to prevent synthetic genome escape [100], [101], [102] and resistance to viruses [103], [104], [105], [106]. To overcome the challenges posed by complex codon usage, researchers have extensively investigated the recoding of codons.

The replacement of synonymous sense codons can affect various factors, such as gene regulation, ribosome spacing, translation efficiency, and accuracy. Consequently, substituting synonymous sense codons is generally more difficult than substituting stop codons. The development of codon recoding typically involves three stages: (1) the substitution of stop codons to the substitution of sense codons, (2) transitioning from local to global usage, and (3) expanding from bacteria to fungi. This gradual process enhances our understanding of codon usage patterns.

In 2011, Wang *et al.* [107] used multiplex automated genome engineering (MAGE) techniques to simultaneously replace 314 TAG stop codons with TAA in 32 *E. coli* cells, as a means of verifying the feasibility of genome-wide replacements. Following this, Isaacs *et al.* [19] employed hierarchical conjugative assembly genome engineering (CAGE) to gather the replaced codons together. In the end, they successfully constructed four genomes, each containing over 80 modifications, which included all the TAG-TAA codon conversions [19]. For the first time, the feasibility of genome-wide codon recoding has been validated. The researchers then concentrated all their TAG-TAA codon replacement efforts into a single *E. coli* cell to complete the construction of the GRO genome [103]. Napolitano *et al.* [99] utilized coselection multiplex automatable genome engineering (CoS-MAGE) and λ recombination technology to replace 123 arginine rare codons AGR with synonymous codons in all essential genes of *E. coli*. This provides genome-wide experience in performing synonymous substitutions of sense codons [99]. By taking the first steps to extend the technique to human cells, Chen *et al.* [108] have demonstrated the feasibility of recoding and highly multiplex editing in mammalian cells, achieving exceptional base editing by converting TAG to TAA for 33 essential genes with a single transfection.

Researchers have also made numerous attempts at codon recoding from a bottom-up perspective. At first, attempts were made to substitute codons in small sections of the genome or individual genes. To study its codon usage patterns, researchers carried out a limited codon replacement experiment on the *M. mycoides* genome [14]. The researchers recoded 13 uncommon codons within 42 crucial, highly-expressed genes of *E. coli* MG1655 by replacing the original genome sequence, thereby exploring the possibility of genome-wide recoding of sense codons [109]. Venetz *et al.* [110] used chemical synthesis rewriting to rebuild the essential genome of *Caulobacter crescentus*, reducing the number of encoded genetic features from 6290 to 799 through sequence rewriting within the 785,701-bp genome. The process involved introducing 133,313 base substitutions and rewriting 123,562 codons. Using the stepwise integration of rolling circle amplified segments technology (SIRCAS), the researchers iteratively replaced 1,557 leucine codons within 200 Kb of the *Salmonella typhimurium* LT2 genome [111]. This represents the first instance of large-scale, single-type codon replacement work being conducted outside of *E. coli.* However, extending codon replacement work to the genome-scale may still pose numerous challenges. These include the need to consider the effects of codon replacement on the secondary structure of mRNA and ribosome binding [99], as well as its impact on mRNA levels [112]. Substitutions of codons that are synonymous may also result in RNA toxicity [113]. These factors may prevent the widespread promotion of synonymous codon replacement work on a large scale. In 2016, Ostrov *et al.* [20] designed and synthesized genome fragments of *E. coli* with only 57 codons. They then tested the biological function of each recoded genome fragment, thus accumulating valuable experience for genome-wide codon recoding work. In the same year, Wang *et al.* [114] developed an efficient method for replacing the genome of *E. coli*. Then, in 2019, they successfully reduced the 64 codons in the entire genome of *E. coli* to 61 through artificial synthesis and replacement. This was the largest codon recoding effort to date [21].

Eukaryotic genomes are more complex, and therefore codon recoding for eukaryotes presents more challenges. In 2011, Dymond *et al.* [96] designed the right arm of chromosome IX of synthetic *S. cerevisiae*. They replaced part of the chromosomal intergenic sequence with synonymous codons and substituted the stop codon TAG with the synonymous codon TAA. This marked the first attempt at the large-scale substitution of synonymous codons in yeast. Synthetic genomes can also leverage codon degeneracy to eliminate certain restriction sites and adjust the GC content of the genome, with the aim of minimizing genome instability [89]. The replacement of synonymous codons in the yeast genome primarily targets non-coding sequences and stop codons [90], [91], [92], [93], [94], [96]. To the best of my knowledge, large-scale replacement of coding genes has not yet been achieved.

### Chromosome number reduction

Chromosomes carry genetic information, and the count of chromosomes is a straightforward characteristic of the genome. In eukaryotes, the quantity of chromosomes varies among different organisms, ranging from a single chromosome in *Myrmecia pilosula* [115] to thousands of chromosomes in *Ophioglossum reticulum* [116]. While the significance of altered chromosome counts for species evolution remains unclear, aneuploidy is widely recognized as a rapid cellular response to adverse environments in both wild and laboratory conditions [117], [118], [119]. Additionally, studies on yeast genomes have revealed that changes in chromosome numbers can occur [120], [121]. Simplifying research on chromosome numbers could open up new avenues for investigating genome evolution and the underlying causes of disease.

In their previous research, the researchers fused chromosomes in yeast to create organisms with a reduced number of chromosomes and tested their viability [122], [123]. In 2018, two Nature studies were published consecutively, in which researchers achieved the simplification of chromosome number in *S. cerevisiae*, and investigated the limits of this simplification [24], [25]. Furthermore, Shao *et al.* [124] successfully transformed a single giant linear chromosome into a ring structure, achieving the complete removal of all telomeres in *S. cerevisiae*. This model provides a valuable tool for studying the significance of telomere existence. Gu *et al.* [125] rearranged the three original chromosomes of *Schizosaccharomyces pombe* and fused them into a single chromosome in various orders. In a study aimed at simplifying chromosomes in mice, researchers fused the two largest chromosomes, I and II [126]. They discovered that there is a limit to the length of chromosomes that can be maintained in cells while still undergoing normal division. Through simplifying chromosomes, researchers have explored the biological significance of maintaining the current chromosome number during genome evolution. This approach has also introduced a novel method for studying the relationship between chromosome structure and function. Recently, SimCells (simple cells), a type of chromosome-free cell, have gained attention from researchers due to their ability to maintain functional cellular machinery and process various genetic circuits [127], [128], [129]. These cells have the potential to expand synthetic biology into new frontiers, serving as minimal cells for studying the fundamental requirements of life or as chassis cells in biomanufacturing.

### Simplifying multi-copy genes

In nature, chromosome and even genome-scale duplication events have occurred, resulting in an increase in the copy number of many genes [130]. The study of multi-copy genes is an important area in the field of life sciences, as this phenomenon may involve multiple biological processes within the cell. And there remain some unresolved issues related to gene copy numbers that require further investigation.

Researchers have confirmed through experiments that in some cases, multi-copy genes can be a manifestation of genome redundancy [131], [132]. Through systematic assessments of the function of a large number of repetitive genes in *S. cerevisiae*, researchers have found that there is a high degree of redundancy in the multiple copies generated by gene duplication [131]. In evolutionary experiments, researchers have observed the spontaneous loss of genes. For example, *Salmonella* acquired a 1.66 Mb genome duplication, but during a short evolutionary process, a large number of genes were lost. After 2000 generations, only 31% of the duplicated genes remained intact [132].

Studies have shown that changes in the length of tandem repeats play a promoting role in genome evolution [133], [134]. Therefore, investigating these tandem repeats might provide valuable insights into the evolution of genomes. In most eukaryotic organisms, the DNA that encodes the large precursor rRNA (rDNA) is present in multiple copies and is tandemly repeated at one or a few chromosomal loci. *Encephalitozoon cuniculi* has one of the smallest genomes in nature and undergoes extreme reduction of its rRNA [135], resulting in unparalleled structural changes to its ribosomes. This result suggests that rRNA could function in a simpler form. In *S. cerevisiae*, a yeast species that contains approximately 150 copies of tandemly repeated rDNA on chromosome XII. Due to the high number of repetitive sequences and the unclear degree of redundancy, simplification is believed to be a challenging task. In the Sc2.0 project, the researchers successfully removed approximately 1.5 Mb of rRNA repeat sequences from chromosome 12 and transferred them to plasmids [93]. Therefore, they were able to successfully manipulate tandemly repeated rRNA sequences. More efforts are required to determine and streamline the redundancy of rRNA.

tRNAs are relatively common multi-copy genes. Generally speaking, tRNA concentration is positively associated with the number of gene copies [136], [137], [138]. There are significant variations in tRNA repertoires between prokaryotes and eukaryotes [139], [140], [141], [142]. Research reveals that the copy number of tRNA genes increases spontaneously [143], [144], [145] or as a result of genome duplication [146]. And macroscopic eukaryotes, including land plants and vertebrates, which possess a vast range of cell types, have independently developed a significant variety of tRNA anticodons [146]. This development is accompanied by high gene redundancy. Several studies have shown that the copy number of tRNA genes can increase spontaneously in response to increased demand [143], [144], [145], [147]. The evolution of tRNA gene sets can occur through the duplication of existing tRNA genes, allowing for the compensation of insufficient supply [145]. The presence of multiple copies of tRNA genes can enhance the growth fitness of strains in unfavorable environments [143], [144]. Through the analysis of 319 bacterial genomes, the researchers were able to reconstruct the evolution of tRNA and observe the dynamic balance between tRNA copy number and codon usage frequency [148].

Below is a list of several studies that have investigated tRNA gene simplification. The researchers conducted a deletion analysis on the five tRNA gene copies that corresponded to the start codon AUG encoding methionine [149]. Researchers have found that strains with reduced tRNA copy numbers remain viable. Similar results were observed for the deletion of different copy numbers of tRNA^Trp^ [150]. In the sc2.0 project, synV removed 20 tRNAs [92], synX removed 24 tRNA genes [91], and other synthetic chromosomes deleted various tRNA genes [88], [90], [93], [96]. Although these deletions were not specifically aimed at simplifying a single tRNA type, they collectively demonstrate the feasibility of simplifying multi-copy tRNA genes. By methodically deleting all tDNAs located on chromosome III in *S. cerevisiae*, researchers created a “tDNA-less” chromosome [151]. In all of the examples mentioned above, the strains remained viable following the deletion of tRNA.

These findings suggest that there may be a complex mechanism in cells that regulates the levels of individual tRNA genes to match cellular demand. The reason why tRNA genes are maintained in multiple copies needs further investigation. In the future, it would be worthwhile to explore the direction of simplifying either a specific type of tRNA or the entire tRNA family.

## Conclusion and future directions

The study of genome simplification is a focal point of both basic and applied research in the life sciences. It has garnered significant attention and has yielded promising results. Currently, the work of genome simplification needs to be thoroughly explored from two aspects. Firstly, we need to consider what should be simplified, and secondly, we need to think about how to simplify it.

To determine what needs to be simplified, we must first consider the underlying causes of complexity. One of the primary contributors to complexity is the size of the genome [152], [153]. This is because a larger genome typically contains more genes and non-coding sequences, which in turn leads to more gene interactions, complex metabolic regulatory networks, intricate genome structures, and a greater likelihood of genomic modifications. However, it's worth noting that genome size alone does not entirely determine the complexity of biological systems or genomes. The genome of *Tmesipteris obliqua* is 50 times larger than the human genome [154], yet it doesn't exhibit complexity beyond that of the human genome. Therefore, various factors affect the complexity of a genome. For instance, a simple repeat sequence called STR is prevalent in the human genome and plays a crucial role in gene expression and the development of biological phenotypes [155]. Thus, the investigation of repetitive sequences or multi-copy genes might serve as a starting point for exploring genome complexity. The genome is the carrier of genetic information. Structurally, there are evident differences in the genome form between prokaryotes and eukaryotes, and the number of chromosomes in different eukaryotes exhibits significant variation. Hence, these phenomena might be indicative of genome complexity. Another notable feature of the genome is the use of synonymous codons. In 2016, the Genome Project-Write was established with the goal of engineering the genomes of high-order eukaryotic gigabases [22]. The objective of the project is to recode the human cell genome and create an ultra-safe, virus-resistant cell line derived from humans for drug production [156].

Apart from proposing specific simplification goals, one of the future directions for simplification work is to establish a quantitative indicator of genome complexity and a more systematic standard [4], [157]. Additionally, simplification work on genomes should be conducted across a wide range of species to establish general rules and develop effective industrial chassis or biological models. The results of human genome sequencing indicate that more than 98% of the genome comprises non-coding sequences [158], [159], posing a significant challenge for genome simplification. Simplifying the genomes of higher eukaryotes could improve our understanding of complex genomes and represent a major area of exploration in the fields of life and health.

Regarding how to simplify, we have discussed a lot above, and I will summarize it here. To begin with, enhancing our capacity to extract genomic information is crucial. The future of development lies in high-throughput tools for genomic data mining, such as CRISPRi, as well as tools for extracting non-coding functions in eukaryotic genomes, such as Hi-C, ATAC-seq, and ChIP-seq. It's important to prioritize the improvement of these technologies. In addition, we need to enhance our genome manipulation capabilities by developing high-throughput tools for genome modification, such as MAGE and CAGE. The progress in efficient genome editing tools has greatly expedited the process of genome reduction. Specifically, site-specific recombinases have been widely employed for making genomic deletions [160], owing to their remarkable efficiency in targeting and deleting specific genomic regions, as well as their compatibility with multiple strains. Currently, multiple CRISPR/Cas-based deletion technologies have been developed for large DNA fragments, including HDR-mediated deletion, recombinase-mediated deletion, prime editing-based deletion [161], [162], and Cas3-based deletion [163], [164]. These technologies allow for efficient and precise deletion of genome segments, even enabling the potential removal of entire chromosomes [165], [166], thereby offering a robust tool for genome-related research. Moreover, computer-assisted genome simplification is becoming increasingly necessary in our current situation, as it can significantly reduce the time and cost associated with trial-and-error processes. Genome simplification for higher eukaryotes is still in its infancy, and the creation of related databases like ENCODE, gnomAD, GWAS, and others can provide essential information support for genome simplification. Lastly, genomics is undergoing a transition from the descriptive phase of genome sequencing and analysis to the chemical synthesis of complete genomes. Since the synthesis of the first tRNA gene in 1972, advancements in nucleic acid synthesis technology have led to continuous and exponential improvements in both the size of synthetic DNA that can be produced and the accuracy of its sequence. The exploration of genome synthesis in viruses, bacteria, fungi, and humans has yielded valuable experience [85], enhancing our understanding of genome sequences and enabling the formulation of more rational principles for genome reduction. Additionally, the decreasing cost and improved capabilities of genome chemical synthesis allow for rapid completion of genome synthesis and expedite the process of genome reduction through the iterative design-build-test-debug-learn cycle. Synthetic chromosome rearrangement technology, such as SCRaMbLE, offers a new approach to large-scale simplification of synthetic chromosomes and represents a crucial direction for future simplification research. Overall, researchers are well-prepared to explore genome simplification, making this an opportune moment to delve into this topic.

## Compliance with ethics requirements

This article does not contain any studies with human or animal subjects.

## CRediT authorship contribution statement

**Xiang-Rong Chen:** Investigation, Visualization, Writing – original draft. **You-Zhi Cui:** Conceptualization, Supervision, Writing – review & editing. **Bing-Zhi Li:** Conceptualization, Supervision, Project administration, Funding acquisition, Writing – review & editing. **Ying-Jin Yuan:** Conceptualization, Supervision, Project administration.

## Declaration of Competing Interest

The authors declare that they have no known competing financial interests or personal relationships that could have appeared to influence the work reported in this paper.
